# Exploring Nurse and Patient Experiences of Developing Rapport During Oncology Ambulatory Care Videoconferencing Visits: Protocol for a Qualitative Study

**DOI:** 10.2196/27940

**Published:** 2021-06-14

**Authors:** Paula D Koppel, Jennie C De Gagne

**Affiliations:** 1 Duke University School of Nursing Durham, NC United States

**Keywords:** nursing, oncology ambulatory care, provider-patient relationship, rapport, telehealth, videoconferencing visits

## Abstract

**Background:**

Telehealth videoconferencing has largely been embraced by health care providers and patients during the COVID-19 pandemic; however, little is known about specific techniques for building rapport and provider-patient relationships in this care environment. Although research suggests that videoconferencing is feasible and can be effective for some types of care, concerns about the impact of technology on provider-patient relationships exist across health disciplines. Suggestions for adapting some in-person rapport techniques, such as the use of small talk, eye contact, and body language to facilitate trust, personal connection, and communication during videoconferencing encounters, have been discussed in the popular press and clinical commentaries. Notably, evidence regarding the effects of these strategies on rapport and clinical care outcomes is lacking. Understanding how to establish rapport in videoconferencing visits is especially important in oncology nursing, where rapport with patients enables nurses to become a source of emotional support, helping patients adapt and navigate the cancer journey.

**Objective:**

This study aims to investigate the nature of nurse-patient rapport in ambulatory cancer care videoconferencing visits. The objectives include exploring how patients with cancer and nurses describe experiences of rapport and strategies for cultivating rapport in videoconferencing visits and similarities and differences identified by patients with cancer and nurses between experiences of rapport in videoconferencing and in-person visits.

**Methods:**

Semistructured narrative interviews of patients with cancer and nurses will be conducted to understand the experience of rapport building in videoconferencing visits. Nurses and patients will be interviewed separately to facilitate an understanding of the perspectives of both types of participants. Interviews will be conducted on a secure videoconferencing platform. This qualitative descriptive study will describe participant experiences in a manner that, although not without interpretation, is as close to the data as possible. The research team will meet regularly to discuss, define, and document codes, categories, and themes, and the team will maintain a detailed audit trail of analytical decisions. In addition, member checking will enhance the rigor of the study. Nurse and patient interviews will be analyzed separately using identical procedures and may be explored side by side in the final analysis to provide a comparative analysis. Data management and analysis will be performed using NVivo 12.

**Results:**

Data collection will begin during summer 2021, with results from the data analysis anticipated by winter 2021. A research team trained in qualitative methodology will use conventional content analysis to analyze the data using first- and second-level codes derived directly from the transcribed text data.

**Conclusions:**

This study aims to determine what behaviors, communication techniques, and relational practices need to be adapted in videoconferencing telehealth visits, setting the foundation for future development of interventions and evidence-based practice guidelines for relationship building during videoconferencing telehealth visits.

**International Registered Report Identifier (IRRID):**

PRR1-10.2196/27940

## Introduction

### Background

When the replacement of ambulatory in-person visits with videoconferencing suddenly became essential for persons with cancer because of the COVID-19 pandemic, providers had little experience or existing research to support this change in practice [1,2]. Some oncology ambulatory care centers went from all in-person visits to more than 50% of patient visits on videoconferencing [3]. Although videoconferencing has been broadly embraced by providers and patients during the pandemic, little is known about specific techniques for building rapport and provider-patient relationships in this care environment.

Review studies of videoconferencing in telehealth, although often focused on feasibility and acceptability for patients who are geographically underserved, show that this computer-mediated modality has utility and even comparable outcomes with in-person care for patients with a variety of chronic diseases and mental health challenges [4-7]. A systematic review of 15 studies conducted in oncology suggests that videoconferencing is feasible and can be effective in the care of some cancers [8]. More recent studies have focused on videoconferencing for palliative care consultation and support to patients, family caregivers, and community-based care providers [9-11]. These studies indicate that videoconferencing is feasible and often preferable for palliative care consultations [11,12], hospice family meetings [13,14], and support groups [15].

### Knowledge Gaps

Although research suggests that videoconferencing is feasible and can be effective for some types of care, concerns about the impact of technology on provider-patient relationships exist across health disciplines. Nurses, physicians, and mental health providers have expressed concern that the two-dimensional interactions in video conferencing coupled with the loss of physical proximity, presence, and touch depersonalizes care and inhibits the providers’ ability to best understand the patient and demonstrate care [16-19]. In palliative care studies, providers have indicated a reluctance to initiate emotional topics, feeling a need to be physically present with the patient to provide necessary support [20,21]. Reluctance has also been described because providers cannot be sure that patients have adequate privacy in videoconferencing visits [14]. Suggestions for adapting some in-person rapport techniques such as the use of small talk, eye contact, and body language to facilitate trust, personal connection, and communication during videoconferencing encounters have been discussed in the popular press and clinical commentaries [1,22-24]. However, few studies seem to have evaluated these modalities [25-28] or more advanced relational skills such as presence [29], conveying caring [30], empathy [31], and person-centered care [32]. Even in telepsychology, where research is more robust [33], providers remain concerned about the impact of videoconferencing on therapeutic alliance and nonverbal communication channels [16,34] and are uncertain about how best to adapt techniques.

### Importance of Nurse-Patient Rapport

Rapport has been defined as a connection established with another person based on respect, acceptance, empathy, and a mutual commitment to engagement [35,36]. Interpersonal interventions that cultivate rapport between patients and providers have the potential to improve patient health outcomes and satisfaction [37,38]. For persons with cancer, feeling known personally and connected with nurses and health care providers on a level beyond their disease process reduces suffering and improves satisfaction, health outcomes, and quality of life [39-41]. Being known beyond their disease includes acknowledging the roles that patients have outside of being a person with cancer, including their personal and professional roles, and interacting with them as people rather than as patients [39,42]. Research suggests that rapport makes a trusting and therapeutic relationship more likely [43,44] and, in turn, enables providers to become a source of emotional support, helping patients adapt and navigate the cancer journey [35,45-47]. The cancer journey is often a prolonged experience that spans diagnosis, treatment, and years of survivorship with ongoing care and multiple comorbidities [48]. Having a nurse who is not only knowledgeable but who can also provide a whole-person approach to care is essential. Ambulatory oncology nurses play a pivotal role in a patient’s cancer journey by helping patients and families integrate new information and build the capacity and skills to adapt and address care challenges [47,49]. For example, a patient with cancer who lives alone or has a baseline mobility impairment may require a very different plan of care to successfully manage common side effects such as nausea, vomiting, and diarrhea. As such, nurse-patient rapport facilitates the trusting relationship necessary to ensure holistic assessment of needs, personalization of care, and adaptive work [39,50-52].

### Critical Need for Research

Studies suggest that relationship development and communication in videoconferencing encounters, although similar to in-person interactions, present unique challenges that can affect rapport, diagnostic accuracy, and treatment compliance when not addressed [25,28]. For example, camera placement and the ability to visualize the self during videoconferencing platforms create both a downward gaze [53] and excessive levels of gaze [54] that are unnatural when compared with in-person encounters. In addition, video and audio lapses that result in overlapping conversations or uncomfortable periods of silence interfere with communication [28]. Studies comparing in-person and videoconferencing encounters have found that providers use less empathetic, supportive, and facilitating statements [31] in virtual encounters, and there is less information exchange, with the presentation of fewer problems [55]. Of note, evidence regarding the effects of these findings on rapport and clinical care outcomes is lacking.

### Research Aim and Questions

The purpose of this qualitative descriptive study is to explore the nature of nurse-patient rapport in ambulatory cancer care videoconferencing visits. The use of videoconferencing in telehealth nursing is relatively new, especially in oncology ambulatory care. With little existing research to build on, prioritizing studies that provide a foundation for future inquiries is essential. This gap requires a descriptive, exploratory study to increase our understanding of nurse-patient rapport in such a context. Knowledge gaps include an understanding of the attributes of videoconferencing experiences for nurses and patients, along with antecedents, facilitators, barriers, and outcomes. The proposed study will address (1) how patients with cancer and nurses describe experiences of rapport and strategies for cultivating rapport in videoconferencing visits and (2) similarities and differences identified by patients with cancer and nurses between experiences of rapport in videoconferencing and in-person visits.

## Methods

### Overview of the Study Design

Nurses and patients will be interviewed separately to ensure that both can speak freely about their experiences and to facilitate understanding of the perspectives of both types of participants. Semistructured interviews will be conducted on Zoom (Zoom Video Communications), a secure videoconferencing platform. Narrative interviewing guidelines will be used, providing participants with the opportunity to describe their experiences before the researcher asks any probing questions. This approach aligns with our qualitative descriptive methodology by focusing on how participants describe their experience of rapport using everyday language to provide insight on videoconferencing [56]. As suggested by Sandelowski [56,57], a descriptive qualitative approach focuses on participants’ descriptions of their experiences while limiting the interpretation of meaning to only what is directly reported by the participant. Unlike a phenomenological approach, which deeply explores a few homogenous participants’ lived experiences, this study will include enough participants to comprehensively describe the experiences of nurse-patient rapport in the new context of videoconferencing in ambulatory oncology [56,57]. Qualitative descriptive research provides rich, in-depth descriptions of experiences that are not feasible with quantitative approaches [58]. This study aims to uncover patterns and themes concerning how rapport is experienced during videoconferencing visits to provide a foundation for the development of practice guidelines.

### Participants and Setting

The study recruitment, consent process, and data collection will be conducted remotely because of COVID-19 restrictions. Stratified purposive sampling will be used to recruit participants from an academic ambulatory oncology center in the northeastern United States. Providers at this center have had recent experiences with videoconferencing visits to provide rich data about these encounters. Stratification will include the number of telehealth videoconferencing visits that patients and nurses have experienced. The literature suggests that the level of experience influences ease of use and perceptions of telehealth videoconferencing visits [4]. Patients will be stratified into 2 groups (ie, ≤2 and >2 videoconferencing visits). Nurses will be stratified into 2 groups (ie, 10-20 and >20 videoconferencing visits). Further stratification may be added to ensure that the sample is diverse, with various perspectives from participants of different ages, backgrounds, and experiences. Participants will be asked to share self-identified information, including age, gender identity, race, ethnicity, household income (patients), education, length of time receiving care (patients) or duration of employment (nurses) at the ambulatory care clinic, and the estimated number of telehealth videoconferencing calls that they have participated in as patient or nurse during the last 12 months. The exact size of the final sample will depend on data saturation in each of the 4 stratified groups and be large enough to capture the rich experiences of nurses and patients but small enough to permit a thorough analysis of the data [59]. Recruitment will end when no new themes related to the research questions are identified, indicating data saturation [60]. Participants who do not have access to technology and adequate broadband width to support videoconferencing will be excluded from the study. As this proposed study aims to analyze the experiences of individuals who have this technology and experience, future studies that will focus on patients with limited technology access are planned.

### Inclusion and Exclusion Criteria

The inclusion criteria for patients are (1) adults (aged ≥18 years), (2) able to read and converse in English, (3) receiving care and undergoing active treatment at the identified oncology ambulatory care center, (4) have participated in at least one videoconferencing visit with a nurse from the identified oncology ambulatory care center within 3 months before the interview, and (5) enrollment in the oncology ambulatory care center’s secure web-based platform with the necessary technology to conduct the videoconferencing interview. Patients with a medical diagnosis related to cognitive impairment (eg, Alzheimer disease or related dementias) will be excluded. The inclusion criteria for nurses are (1) licensed registered or advanced practice nurses employed at the oncology ambulatory care center for at least a year after orientation and (2) participation in videoconferencing visits with patients at the identified oncology ambulatory care center.

### Ethical Considerations

The institutional review board (IRB) application for this study is currently under review. The Center for Research in Nursing and Patient Care Services within the cancer center supports the facilitation of this study. The applicant’s university IRB will also review the proposal after it has been approved by the cancer center. Approval from both participating IRBs will be received before beginning the study. Written informed consent will be obtained from all participants before data collection.

The research methods, including qualitative interviews and asking for demographic information, involve minimal risk to participants. A study information sheet, which includes the study purpose and activities as well as the primary researcher’s contact information, will be provided electronically to participants. This will allow participants to contact the research team directly. This information will also be shared verbally during the consent process and included in the informed consent form. Participants will be informed that their participation is voluntary and can be stopped or rescheduled at any time. Participants will also be informed that they can withdraw from the study at any time without any impact on their care or employment. The researchers will maintain open and honest communication throughout the process and create an environment of trust and safety by answering all questions and allowing participants as much time as necessary. Patients will be allowed to include their informal caregivers in this process because the literature suggests that such inclusion may reduce the burden of the recruitment process for patients with cancer [61]. However, informal caregivers will not be interviewed and will be asked to allow the patient to answer all the interview questions independently.

Care will be taken to contextualize any data that could potentially threaten the confidentiality and privacy of participants. For example, exemplar quotations will simply refer to participants by number or pseudonym when presenting the results. Data collected from participants will be deidentified and used only for research purposes and associated dissemination. Participants will be assigned unique identification numbers that will be used on all data collection forms and data files (ie, interviews, transcripts, and demographic questionnaires). Electronic files, including the key for the unique identification numbers and corresponding participant names, will be stored on the researchers’ computers on a secure password-protected hard drive or server with firewall protection and multifactor authentication. Any hard copies of transcripts or field notes will be stored in a double-locked location. Only members of the research team will have access to the electronic and printed files. Interviews will be conducted on Zoom, the institution-approved secure videoconferencing platform with approved data management and security features that allow secure recording and storage without recourse to third-party software. Zoom security measures include user-specific authentication, real-time encryption, and the ability to back up recordings to the aforementioned secure password-protected server.

Participation in this study offers no direct benefits to the nurses or patients who participate. However, participants may find it beneficial to discuss and reflect on their encounters and the nurse-patient relationship. They may also feel a sense of satisfaction from contributing to the advancement of knowledge and future practice implications attributed to the study. Nurses and patients will be invited to attend the presentation of the study results. Evidence demonstrates that both patients with cancer [61] and nurses [62] often gain a sense of fulfillment when participating in research.

### Recruitment

Conducting interviews on the institution-approved platform will allow recruitment and data collection to proceed even as social distancing is maintained during the ongoing pandemic. Recruitment of participants will be carried out at the oncology clinics at the ambulatory care center where nursing videoconferencing is currently part of the care process. Nurses and patients will be recruited through a combination of efforts, including announcements at nursing staff meetings, postings on a web-based study recruitment message board, and the distribution of study brochures. Nurses will be recruited into the study first, and they will be invited to share the study information sheet with eligible patients. Interest in, and support for the study has been obtained from the cancer center’s nursing leadership, who will foster opportunities for the research study to be presented to nursing staff. Nurses will self-identify and be asked to share the study brochures with patients who may be potential participants in the study. Information about the study in web-based research bulletins will also allow patients to self-identify. Participants interested in participating in the study will be screened and provided information on the study and informed consent by the researchers through telephone or email. A time for the interview will also be agreed upon. The written consent and demographic data collection will be carried out through Research Electronic Data Capture at the time of the interview. All patient and nurse interviews will be conducted on the institution-approved videoconferencing platform to ensure a secure encounter. Patients will be allowed to include their informal caregivers in this process because the literature suggests that this reduces the burden of the recruitment process for patients with cancer [61]. Although the concern for adding burden to patients with cancer is legitimate, evidence suggests that patients with cancer often find meaning in the disease process by participating in research [61,62]. No subjects will be excluded from the study based on age, self-reported gender identity, race, or ethnicity, except where necessary to meet the inclusion and exclusion criteria.

### Data Collection Procedures

Individual nurses and patients will be interviewed separately. Interviews will be conducted on Zoom, the institution-approved platform, which is used by the ambulatory care center. Using the institution-approved platform will ensure that the participants are familiar with the technology and will also prompt memories of their experiences of rapport in videoconferencing encounters. Videoconferencing has been shown to be a feasible and acceptable vehicle for collecting qualitative data, with both researchers and participants reporting high levels of satisfaction [63], and a comparison of in-person interviews with videoconferencing interviews found little difference in the development of interviewer rapport [64]. Interviews will be audio recorded and transcribed to allow analysis of these text-based data. Data analysis will be ongoing and occur concurrently with data collection.

Interview guides for nurse and patient participants are under development by the research team to direct the semistructured interviews. Seminal research by Radwin [46] and Thorne [41] on the perceptions of patients with cancer of clinical care are guiding the development of the interview guide. The interviews will use a narrative format with 4 phases: initialization, main narrative, questioning, and closure [65]. During the initialization phase, participants will be asked to confirm their understanding that the interview is being recorded. The purpose of the interview will be described as, “This is an opportunity to share your experiences of having videoconferencing visits with your oncology nurse.” A grand tour question will be used to initiate the interview: “Please describe your thoughts and feelings about having visits with your oncology nurse via videoconferencing.” Per narrative interviewing guidelines, participants will be given an opportunity to tell their complete story (main narrative phase) about their experiences before the interviewer asks any probing follow-up questions [65]. In the questioning phase of the interviews, participants will be asked to describe their experiences of rapport in nurse-patient interactions during both in-person and videoconferencing encounters. Follow-up questions will be used to probe more deeply after the initial responses to provide rich, detailed descriptions. For example, participants will be asked to reflect on how experiencing rapport in videoconferencing visits was similar to, or different from, their in-person experiences. In addition, a broad question will be used to explore whether the participants felt that strategies or contextual issues (ie, technology challenges, two-dimensional nature of the relationship, ability to detect facial features or body language, lack of touch or other sensory stimulation) may have affected their experience of rapport in videoconferencing visits either positively or negatively. Questions for nurses and patients, although similar, will be slightly modified, given their differing roles. Before concluding, the interviewer will ask participants if there is anything else that they want to share. The guide for patients can be found in Textbox 1.

**Patient interview guide.**

**Grand Question (Start Here): Please Describe Your Thoughts and Feelings About Having Videoconferencing Visits With Your Nurses**

- Follow-up probing questions
  - How would you describe rapport?
  - How do you experience rapport with your nurse in a videoconferencing visit? For example, what does it look like and how does it feel? How does it feel and look when you do not have rapport with your nurse?
  - How do you go about developing rapport with your nurse in a videoconferencing visit?
  - How does the experience of rapport with your nurse in a videoconference visit compare with a traditional in-person encounter? What are the differences in your visits when videoconferencing is used? Is it easier to establish rapport in-person?
  - How does having a videoconference visit affect your ability to communicate with your nurse?
  - What factors do you think may be influencing or have an impact on your ability to build rapport with your nurse in videoconferencing visits?
  - Would you want to continue videoconferencing visits once the COVID-19 pandemic is controlled? Why or why not?
  - Is having rapport important to you and if so, why or why not?
  - Is there anything else you would like to share with me?

### Data Management and Analysis

#### Overview

Conventional content analysis will be used to analyze the qualitative data collected in this study. This approach is useful in exploring areas where little is known and robust descriptive data are needed to better understand the research questions [66,67]. The purpose of this study is to understand how rapport is described and evolves within the context of this new clinical setting by focusing on manifest content rather than symbolic meaning (latent content). By focusing on manifest content, the analysis is firmly placed in the realm of content analysis rather than thematic analysis or other approaches, such as narrative, discourse, or semiotic analysis [67,68]. Codes will be derived directly from the transcribed text data, keeping the coding close to the participants’ descriptions [56,67]. Nurse and patient interviews will be analyzed separately using identical procedures. The content analysis management process described by Elo and Kyngäs [66] will be used to organize the analysis process. This management process includes 3 phases: preparation, organization, and reporting.

#### Preparation Phase

With each interview representing the unit of analysis for this study, data preparation will include reading through each entire transcript while listening to the entire interview and noting important topics in the margin. The data preparation phase allows for immersion in the text data and for the researchers to become familiar with each case as a whole [66]. After reading each transcript, a summary analytic memo will be initiated to capture overall impressions, a holistic view of the interview, contextual information that might have influenced the interview, and any personal perceptions [69]. The data preparation phase will also make transparent any research team member beliefs or experiences elicited by the data that require bracketing [69].

#### Organizational Phase

The goal of the organizational phase is to label and condense the data into meaningful units, allowing patterns and relationships within the units to emerge. Initially, research team members will go line by line through the transcript, applying meaningful codes [67]. An inductive approach that allows the codes to emerge from the data will be used. Coding is often divided into 2 levels [69]. First-level coding methods assign codes to data units as they are read line by line again. There are many first-level coding methods, but based on this study’s aim to capture the interpersonal experiences of nurses and patients who participate in videoconferencing visits, the coding method will likely involve both process and emotional coding [69]. Process coding captures interactions and outcomes, whereas emotional coding is useful for descriptions of intrapersonal and interpersonal experiences [69]. In addition, codes using the participants’ own words, also called in vivo coding, may provide rich labels that are authentic to participants’ experiences [69]. To support the exploration of the data, team members will consider codes that answer questions (ie, what, who, how, when, where, why), capture actions (eg, watching, listening, advocating), and describe characteristics of the experience (eg, supportive, encouraging, warm, interested). This first-level coding approach is purposefully selected to represent the manifest content of the data rather than its symbolic meaning [69].

Second-level coding will explore relationships and patterns in the data, resulting in categories and themes [66]. This process will be intentionally iterative to keep the analysis as close as possible to the participants’ accounts, encompassing the experiences of all the participants interviewed, with ideas emerging to either confirm data already analyzed or provide new data that need verification [56,70]. Analyses will include creating, defining, and recording codes, categories, and themes and matching the themes with exemplar quotations. After separate analyses of patient and nurse data have been completed, a comparison of the data will allow the exploration of similarities and differences. This analysis may result in additional themes for the final reporting phase [66].

Code names, definitions of codes, and exemplar quotes will be constructed in a codebook using the data analysis management tool NVivo 12.0 (QSR International Pty Ltd). During the initial weeks of the analysis, the authors will individually code the same cases and meet as a team to compare and define codes. Once all research team members agree on the coding of 20% of the transcripts, a codebook will be created to guide coding of the remaining transcripts. The team will continue to meet to discuss new codes and revise the codebook as needed. As the codebook evolves and agreement between team members becomes consistent, team members will code different cases. Bimonthly scheduled coding meetings will be used to discuss and clarify the coding of uncertain data segments. Suggested codebook revisions will always be discussed until an agreement is reached. Changes will be documented in analytical memos to ensure documentation of the process and clear, consistent data coding. Any revisions in codes will require revisiting the previous coding to ensure the integrity of the analysis.

#### Reporting Phase

The final phase of the analysis process is reporting. Exemplar quotes from the participants will be used as evidence of the findings [66,67]. As nurse and patient interviews will be analyzed separately, data reporting will reflect themes for each group separately but may be presented side by side to illustrate a comparative analysis. The findings will be evaluated within the context of related theories and evidence-based research [66,67]. A narrative of the final analysis will create a story of the data, adding insight and new knowledge [66,71].

### Plan for Ensuring Rigor or Trustworthiness of Findings

The study’s rigor, also described as trustworthiness in qualitative research [60,70], will be enhanced by (1) conducting all analyses as a research team (ie, a nursing doctoral student well versed in qualitative research and a nurse faculty researcher with 15 years of qualitative research experience), with weekly coding meetings to discuss and define all codes, categories, and themes; (2) collecting and analyzing the data concurrently, listening carefully while remaining open to the emergence of unexpected findings, and being willing to let go of poorly supported ideas [70]; (3) using detailed memos to create an audit trail of analytical decisions [60,72]; (4) confirming that the categories represent expansive and diverse experiences with exemplar quotations from multiple participants [60,73]; and (5) using member checking techniques by asking participants clarifying questions during the interviews [60]. The Consolidated Criteria for Reporting Qualitative Research checklist will be used to guide the reporting of the study results [74].

Risks for bias in general include the effects of the researcher on the participants; the effects of the participants on the researcher; and the researcher’s own perspectives, experiences, assumptions, values, and beliefs. More specific risks for bias include the influence of COVID-19 as the context that increased the use of videoconferencing, level of experience of the participants with videoconferencing, and the use of nurses to help with recruitment. In qualitative research, the researcher is the instrument [60], and this is reflected in the strategies used to enhance the rigor and trustworthiness described previously. In large measure, rigor will be enhanced with reflexivity strategies that include ongoing memos with researcher self-reflections. This transparency will also enhance the research team’s capacity to remain receptive to new emerging findings during the analysis process [60]. The interview guide, developed by the researchers, is based on a systematic review of the literature, and the narrative interview approach involves starting with a grand question that is designed to allow the participant’s experience to guide the interview. In addition, stratified purposive samples will be used to ensure collection of data from participants who have multiple perspectives, and the nurses who assist in recruitment will be asked to offer the study information to all their patients who meet the study criteria. This will reduce the risk of nurses only telling a particular group of patients about the study (ie, patients with whom they share a good rapport). Finally, a presentation of the findings will be set within the context of the pandemic.

## Results

### Study Status

IRB approval will be obtained before beginning data collection. Data collection will begin during summer 2021, with analysis expected to be completed by winter 2021.

### Anticipated Results

Few studies have focused exclusively on rapport building in videoconferencing [21]; however, rapport is often mentioned in research conducted on telehealth videoconferencing visits. These incidental findings provide some clues to our expected results. For example, studies often highlight how the visual component of videoconferencing makes relationship building easier compared with telephonic consultation [21,75-77]. Other studies suggest that adjustments to the background, camera positioning, and volume, along with the increased use of verbal confirmations during the interaction, are useful [21,29,75,78]. Helping patients navigate technology with a positive attitude, including the use of in-person support to provide the caring touch and hands-on technical assistance, has been reported to enhance rapport [29,54,79,80].

Unique barriers to rapport in videoconferencing visits have also been reported as secondary findings, and they include uncertainty around patient privacy [9,12,21,79], loss of sensory input owing to the limited view of the peripheral space and full visualization of body language [14,54,81], loss of physical connectedness [21,29,79], and patients being left out in provider exchanges [82,83]. Finally, technology failures have been described as having a negative impact on rapport [29,81].

By focusing our exploration exclusively on patient-nurse rapport in oncology ambulatory videoconferencing visits, this study seeks to fully describe this experience from both nurse and patient perspectives. The study will also seek to understand the unique challenges, facilitators, and barriers to developing and experiencing rapport in these computer-mediated encounters. This investigation may validate some of the incidental findings from other studies or uncover new considerations.

## Discussion

### Study Significance

A strong and supportive nurse-patient relationship is especially important for persons with cancer. Looking at how the movement toward videoconferencing visits during the COVID-19 pandemic affects the nurse-patient relationship and the capacity to maintain high quality, supportive cancer care is essential, given the likelihood that telehealth videoconferencing visits will become an enduring component of cancer care. Although caring within the videoconferencing technological environment may require adapting our practices, it must not detract from the essential nature of nursing. One view of caring in a technological medium is described by Locsin and Purnell [19] in their theory, Technological Competency as Caring in Nursing. This midrange theory views technology as a complementary opportunity that can facilitate knowing and connection [19]. The nurse’s technological competency is seen as another way of caring [19,84]. From this vantage point, videoconferencing visits can be a way to maintain human connectedness during the pandemic or even beyond. Technological Competency as Caring in Nursing describes the human connection and communication between the nurse and patient as a *cocreated moment* essential to protecting humanness and preventing patients from becoming objects of care in technological environments [19].

### Conclusions

The pandemic makes this exploration of rapport in telehealth videoconferencing with nurses and patients timely. The rapid and successful use of videoconferencing visits, coupled with potential benefits to patients, providers, and health care systems, suggests that patient care using this technology will likely continue to be a significant component of oncology ambulatory care even after the pandemic has subsided [2,85-87]. This research will help determine what behaviors, communication techniques, and relational practices need to be adapted to advance effective nurse-patient rapport in oncology videoconferencing visits. This study will set the foundation for developing interventions and evidence-based practice guidelines for developing a nurse-patient therapeutic relationship during videoconferencing telehealth visits.

## Abbreviations

IRB: institutional review board
